# Grapevine Badnavirus 1: Detection, Genetic Diversity, and Distribution in Croatia

**DOI:** 10.3390/plants11162135

**Published:** 2022-08-16

**Authors:** Martin Jagunić, Alfredo Diaz-Lara, Maher Al Rwahnih, Darko Preiner, Kristian Stevens, Goran Zdunić, Minsook Hwang, Darko Vončina

**Affiliations:** 1 Department of Plant Pathology, Faculty of Agriculture, University of Zagreb, 10000 Zagreb, Croatia; 2 School of Engineering and Sciences, Tecnologico de Monterrey, Campus Queretaro, Queretaro 76130, Mexico; 3 Department of Plant Pathology, Foundation Plant Services, University of California-Davis, Davis, CA 95616, USA; 4 Department of Viticulture and Enology, Faculty of Agriculture, University of Zagreb, 10000 Zagreb, Croatia; 5 Centre of Excellence for Biodiversity and Molecular Plant Breeding, 10000 Zagreb, Croatia; 6 Departments of Computer Science and Evolution and Ecology, University of California-Davis, Davis, CA 95616, USA; 7 Institute for Adriatic Crops and Karst Reclamation, Put Duilova 11, 21000 Split, Croatia

**Keywords:** conventional PCR, real-time PCR, autochthonous and introduced grapevine varieties, sequencing, phylogeny

## Abstract

Grapevine badnavirus 1 (GBV-1) was recently discovered in grapevine using high throughput sequencing. In order to carry out large-scale testing that will allow for better insights into virus distribution, conventional and real-time PCR assays were developed using sequences both from previously known, and four newly characterized isolates. Throughout the growing season and dormancy, GBV-1 can be detected by real-time PCR using available tissue, with the possibility of false-negative results early in vegetation growth. GBV-1 real-time PCR analysis of 4302 grapevine samples from the Croatian continental and coastal wine-growing regions revealed 576 (~13.4%) positive vines. In the continental wine-growing region, virus incidence was confirmed in only two collection plantations, whereas in the coastal region, infection was confirmed in 30 commercial vineyards and one collection plantation. Infection rates ranged from 1.9 to 96% at the different sites, with predominantly autochthonous grapevine cultivars infected. Conventional PCR products obtained from 50 newly discovered GBV-1 isolates, containing the 375 nucleotides long portion of the reverse transcriptase gene, showed nucleotide and amino acid identities ranging from 94.1 to 100% and from 92.8 to 100%, respectively. The reconstructed phylogenetic tree positioned the GBV-1 isolates taken from the same vineyard close to each other indicating a possible local infection event, although the tree nodes were generally not well supported.

## 1. Introduction

Grapevine (*Vitis vinifera* L.) is one of the most widespread fruit crops around the world, including Croatia. In addition to the many introduced grape cultivars, viticulture in Croatia is characterized by at least 125 autochthonous cultivars, which are of commercial and cultural importance [1]. The most important step in the revitalization and protection of old cultivars is clonal and sanitary selection, with special attention paid to viruses as vegetatively transmitted pathogens [2,3,4,5].

Viruses are currently one of the greatest challenges in viticulture, primarily because about 30 of them are considered pathogens that cause a wide range of symptoms and shorten the lifespan of grapevines, affecting the quality and quantity of grapes produced [6]. To date, 86 virus species from 18 families and 35 genera have been described as infecting grapevines [7,8]. In the last decade, a significant number of grapevine viruses have been discovered with the advent of high throughput sequencing (HTS), which can detect the virus even in asymptomatic plants by directly ascertaining molecular sequences “in vivo” [9]. Likewise, several badnaviruses have been discovered that had not previously been detected in grapevines. The genus *Badnavirus* belongs to the family *Caulimoviridae*, the only known plant-infecting family of double-stranded DNA (dsDNA) viruses. According to the current taxonomy released by ICTV (https://ictv.global/report/chapter/caulimoviridae/caulimoviridae, accessed on 1 July 2022), the family *Caulimoviridae* is divided into 11 genera comprising 94 species, of which 67 viruses are assigned to the genus *Badnavirus* [10]. Badnaviruses are considered important pathogens of many monocotyledonous and dicotyledonous crops grown mainly in tropical and subtropical areas: banana, black pepper, cocoa, citrus, sugarcane, taro, yam, etc. [11,12]. In addition to vegetative propagation, some representatives are transmitted by seeds, and mealybugs or aphids in a semipersistent manner [13].

The first badnavirus infecting grapevine was discovered in 2011 in the United States of America (USA) using HTS and named grapevine vein clearing virus (GVCV) [14]. The virus causes vein clearing and vine decline, a severe disease that has led to uprooting of many vineyards in the Midwestern USA [15]. GVCV is transmitted by aphids and alternative hosts, besides grapevine, have been identified: *Ampelopsis cordata* and *V. rupestris*. This virus has been currently confirmed only in four USA states [16,17]. Despite its low prevalence, GVCV, along with grapevine Pinot gris virus (GPGV) and grapevine red blotch virus (GRBV), poses a serious threat to the wine and grape industries [18].

The second grapevine-infecting badnavirus was discovered in 2015 in Greece, in the Greek autochthonous variety Roditis and named grapevine Roditis leaf discoloration-associated virus (GRLDaV) [19]. Symptoms of this virus were observed in the early 1980s in central Greece and, at that time, according to symptoms, the disease was named Roditis leaf discoloration (RLD) [20]. Shortly after its discovery, the virus was also confirmed outside Greece: in Italy, Croatia and Turkey [21,22,23]. Vector transmission by vine mealybug (*Planoccocus ficus* Signoret) has been proven [24], as well as mechanical transmission to herbaceous hosts (*Nicotiana benthamiana, N. tabacum, N. rustica, Physalis floridana*) [19]. Due to the spreading risk, the virus is currently on the EPPO alert list [25].

The third badnavirus infecting grapevine was confirmed in Croatia in 2018 by HTS and named grapevine badnavirus 1 (GBV-1) [22]. The virus was discovered in two vines (cultivars Ljutun and Vlaška) with the symptoms of reduced growth, both present at the virus collection in Zagreb, but originating from the Kaštela wine-growing region (central Dalmatia) and with identical GBV-1 sequence. Recently, GBV-1 was detected in four samples of cv. Plavac mali (PMC) from the collection plantation in Split, among grapevine accessions originating from the islands of Hvar, Vis and Brač [26]. The genome of GBV-1 includes three open reading frames (ORFs) encoding for two hypothetical proteins and a polyprotein [22]. Since there are no other publications characterizing GBV-1 and its spread, the aim of this study was to develop robust detection methods based on conventional and real-time PCR that will allow large-scale screening and provide a useful toolkit for grapevine virus diagnostics and certification schemes in Croatia and beyond. Additionally, partial sequencing of diverse GBV-1 isolates found in different locations/vineyards will give better insight into the molecular diversity and phylogeny of the virus.

## 2. Materials and Methods

### 2.1. Development of Real-Time and Conventional PCR Detection Protocols

For the development of robust detection methods based on conventional and real-time PCR, 24 vines were selected from the grapevine virus collection (University of Zagreb Faculty of Agriculture) and total nucleic acids were isolated from the leaf petioles according to the previously described protocol [27]. Later, the rRNA depletion and cDNA library were constructed using TruSeq Stranded Total RNA with Ribo-Zero Plant kit (Illumina, San Diego, CA, USA) according to the manufacturer’s protocol. Constructed libraries were sequenced using the Illumina NextSeq 500 platform (University of California-Davis Genome Center). HTS data were subjected to demultiplication and adapter removal using bcl2fastq Conversion Software (Illumina), and de novo assembly was performed using SPADes [28]. The assembled contigs were compared against the complete non-redundant GenBank virus database using BLASTn and BLASTx, providing the annotation used for viral agent identification. As a result, four newly discovered GBV-1 isolates (Supplementary Table S1), together with the sequence previously reported from cv. Ljutun (NC_055481), were used for the development of primers (including degenerate primers) and probes using Primer 3 (https://primer3.org/webinterface.html, accessed on 5 August 2019) and Geneious 10.2.6 (https://www.geneious.com, accessed on 5 August 2019) programs. Primers and probes were designed based on the highly conserved reverse transcriptase (RT) region using the known and newly characterized GBV-1 genome sequences (Table 1).

To validate and compare the sensitivity and detection capability of real-time and conventional PCR assays, GBV-1-infected grapevine accession from the grapevine virus collection was selected and DNA was isolated in three replicates using the DNeasy Plant Mini Kit (Qiagen, Hilden, Germany) and by the cost-effective glycine-EDTA-sodium method (GES) [29], routinely used for the detection of grapevine viruses. For both extractions 0.1 g of leaf petioles were ground in a mortar and pestle with liquid nitrogen. For DNeasy extraction all further steps were conducted according to the manufacturer’s instructions, while for the GES extraction homogenized material was transferred to 2 mL tubes with the addition of 1.8 mL of the grinding buffer (15 mM Na_2_CO_3_, 34.88 mM NaHCO_3_, 0.5 mM PVP 40, 0.2% bovine serum albumin, 0.05% tween 20, pH 9.6 with acetic acid). The tubes were centrifuged at 13,200× *g* for 10 min and the supernatant was transferred to a new 2 mL collection tube. Then, 8 µL of the extract was added to 100 µL of GES buffer (0.1 M glycine, 50 mM NaCl, 1 mM EDTA, 0.5% Triton X, 1% β-mercaptoethanol, pH 9.0 with NaOH), followed by denaturation in a Mastercycler (Eppendorf, Hamburg, Germany) at 95 °C for 10 min. Finally, for both extraction methods purity and quantity of extracts were verified spectrophotometrically (A260/A280) using a NanoPhotometer P330 (Implen, München, Germany).

Isolated nucleic acids by both methods were used for sensitivity tests by serial dilutions. A series of ten-fold dilutions, from 1 down to 1:100,000 and 1:10,000,000 were prepared for GES and DNeasy extractions, respectively, and real-time and conventional PCR reactions conducted in three replicates. The template for reaction was 2 µL of each dilution in 20 µL of the final volume for real-time PCR and 0.2 µL of each dilution in 10 µL for conventional PCR. Reactions were performed according to below described conditions. Additionally, the efficiency of the real-time PCR assay was evaluated using the results of a standard curve (Applied Biosystems 7500 Software ver. 2.3, Life Technologies Corporation, South San Francisco, CA, USA). Dilution results for conventional PCR were evaluated by gel electrophoresis as described below. Finally, both extraction methods were compared on five GBV-1-infected grapevine accessions with undiluted extracts in three replicates per accession.

The real-time PCR was prepared in a 20 µL reaction volume according to the manufacturer’s instructions: 0.4 µM of each primer, 0.150 µM of probe, 5 µL of TaqMan™ Fast Virus 1-Step Master Mix (Applied Biosystems, Thermo Fischer Scientific, Waltham, MA, USA), 10.6 µL of ultrapure water, and 2 µL of DNA extract as template. Used reaction conditions were previously described [30]: initial activation step 10 min at 95 °C, followed by 40 cycles at 94 °C for 15 sec, and elongation step at 60 °C for 1 min. Reactions were carried out using the Applied Biosystems 7500 Real-Time PCR System (Thermo Fischer Scientific, Waltham, MA, USA).

For conventional PCR, a final volume of 10 µL was prepared using a HotStarTaq DNA Polymerase Kit (Qiagen, Hilden, Germany). Thus, the master mix consisted of 0.5 µM of each primer, 1 µL of 10× buffer, 2 µL of Q-solution, 0.2 µM of dNTP mix, 0.05 µL of HotStart Poly enzyme, 6.05 µL of ultrapure water and 0.2 µL of template DNA. The PCR reaction was performed in a Mastercycler (Eppendorf, Hamburg, Germany) under the following conditions: initial activation step of 15 min at 95 °C, 35 cycles of 30 s at 94 °C, 30 s at 55 °C, 1 min at 72 °C and a final elongation step of 10 min at 72 °C. Visualization of PCR products was performed on a 1.5% agarose gel prepared in a 1× TBE buffer containing one drop of GelRed (CareDx AB, Stockholm, Sweden), in horizontal gel electrophoresis (Bio-Rad, Hercules, CA, USA).

### 2.2. Virus Detection during Dormancy and the Growing Season

To investigate GBV-1-detection by real-time PCR throughout the season, different plant material (young shoots at the beginning of vegetation, petioles of old leaves during vegetation, cortical scrapings during dormancy) were collected from five infected vines of cv. Plavac mali (PMC) during the 2020 season. Samples were collected twice per month from the start of vegetation until the leaf fall period (March–October) and in two additional times during dormancy (November–December), for a total of 16 times. In addition to the five GBV-1 infected vines, one virus-free vine was included as a negative control.

### 2.3. Virus Screening in Collection Plantations and Commercial Vineyards

Following the development of a robust and precise detection based on real-time PCR, large-scale screenings in different grapevine collections and commercial vineyards were conducted. In the summers of 2020 and 2021, three petioles from different parts of the canopy were collected from each vine included in the survey and stored at −20 °C until analysis. A total of 4302 samples (vines) were selected from 88 commercial vineyards and 5 collection plantations from 12 different Croatian counties (Požega-Slavonia, Sisak-Moslavina, Krapina-Zagorje, Zagreb County, city of Zagreb, Istria, Primorje-Gorski Kotar, Lika-Senj, Zadar, Šibenik-Knin, Split-Dalmatia, and Dubrovnik-Neretva; Supplementary Figure S1). The sampling strategy and sample size were adjusted considering the predominant cultivars, especially those considered as autochthonous, and the importance of viticulture in the different counties.

The exact vineyards/collections included in the survey were: grapevine virus collection (196 samples), vines and rootstocks collection (91), two national collections of autochthonous Croatian cultivars at experimental station “Jazbina” (591) (all four managed by University of Zagreb Faculty of Agriculture), collection of autochthonous Croatian and introduced grapevine cultivars in Split (Institute for Adriatic Crops and Karst Reclamation) (105), 16 commercial vineyards in the continental region (441 samples) and 72 commercial vineyards distributed along the coastal region (2878 samples). Most of the collected samples (3549, 82.5%) were considered as Croatian autochthonous cultivars, while 753 (17.5%) samples belonged to introduced cultivars (including rootstocks and cv. Graševina as the variety of uncertain origin).

### 2.4. Direct Sequencing and Phylogenetic Analysis

After the field survey, 50 GBV-1-positive vines originating from 23 different locations/vineyards were selected. From ten locations (Mala Rava, Vela Rava, Imotski, Kaštel Sućurac, Ivan Dolac 1, Ivan Dolac 2, Kaštel Lukšić, Velo Vijelo, island of Vis, and Split-collection plantation) three or four GBV-1-infected vines were selected, from the collection Split six, while from other GBV-1-positive locations one vine per site was selected. For the sequencing, the conventional PCR was performed in a reaction volume of 25 µL using the master mix and conditions as previously described. Sanger sequencing of the 419 bps long PCR products, comprising part of the RT region, was performed in both directions at Macrogen Europe (Amsterdam, The Netherlands). After primers removal, the 375 nts long sequences were reviewed and processed in Bioedit 7.2. [31]. The consensus sequences obtained for each isolate were compared phylogenetically with each other and with the GBV-1 reference isolate VLJ-178. The best model of nucleotide substitution and the construction of a phylogenetic tree using the maximum-likelihood (ML) method with 1000 bootstrap replicates was conducted using the MEGA11 software [32] and GRLDaV isolate NV5 (MT783680) was used for rooting.

## 3. Results

### 3.1. Real-Time and Conventional PCR Detection

**Figure 1:**
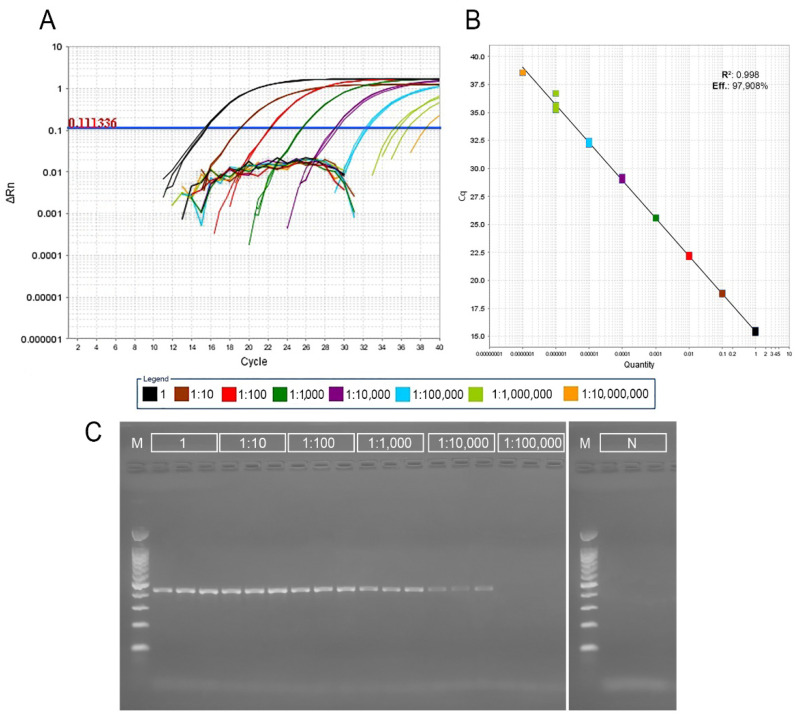
Sensitivity comparison between real-time PCR and conventional PCR assays for the detection of GBV-1 on grapevine accession PMC-313 using three replicates of a 10-fold dilution series. Isolation of DNA was performed using the DNeasy Plant Mini Kit (Qiagen, Hilden, Germany). 1—undiluted extract; 1:10–1:10,000,000—serial 10-fold dilutions. (**A**) Plots of DNA dilution series against threshold cycles values showing the dynamic range of the real-time PCR assay detection. The broken lines below the threshold represents negative controls for each dilution. (**B**) Standard curve analysis of the real-time PCR sensitivity: x-axis—DNA dilution; *y*-axis—measured quantification cycle (Cq) value; R2—determination coefficient; Eff.- real-time PCR efficiency. (**C**) PCR products obtained by conventional PCR on a 1.5% TBE agarose gel; M-marker (GelPilot 100 bp Plus Ladder, Qiagen, Hilden, Germany), N—negative controls for undiluted extract.

The real-time PCR assay with nucleic acids extracted with the DNeasy Plant Mini Kit showed to be accurate and precise. In the serial dilution test performed on the grapevine accession PMC-313 positive results were obtained down to the dilution of 1:1,000,000 for all three replicates per dilution, while the final dilution 1:10,000,000 provided a positive signal just in one out of three replicates. The corresponding standard curve analysis showed that the efficiency of the real-time PCR reaction was 97.908%, with a coefficient of determination of 0.998. Gel electrophoresis of conventional PCR showed a clear amplicon signal of approximately 419 bps in size down to the dilution of 1:10,000 (Figure 1).

The GES extraction performed on the same grapevine accession (PMC-313) showed that the real-time PCR assay was able to detect a signal down to a dilution of 1:100,000, whereas gel electrophoresis of the conventional PCR showed a clear amplicon signal down to a dilution of 1:100. Standard curve analysis showed a real-time PCR reaction efficiency of 107.279%, with a coefficient of determination of 0.997 (Supplementary Figure S2A–C). Finally, to compare the suitability of cost-effective GES extraction vs. DNeasy Plant Mini Kit, the comparison was performed on five infected grapevine accessions in three replicates each. The presence of GBV-1 was confirmed in all five vines by real-time PCR using both extraction methods. The difference in Cq values for DNeasy extraction ranged from 13.1 to 15.2 units, while the GES method performed on same five grapevine accessions resulted in Cq values ranging from 20.3 to 30.5 units (Supplementary Figure S2D). According to the results, real-time PCR with GES extraction was selected as a method suitable for large-scale screening for the presence of GBV-1 with the threshold set up on 31.

### 3.2. Virus Detection during Dormancy and the Growing Season

**Figure 2:**
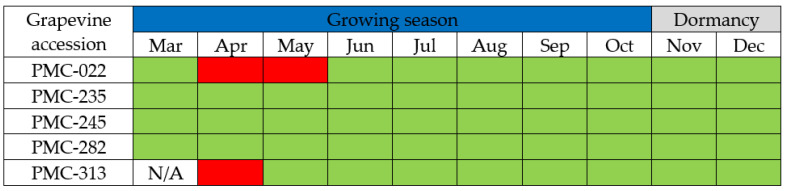
Detection of GBV-1 in five grapevine accessions by real-time PCR during the 2020 growing season and dormancy. Positive results are shown in green, while negative results are shown in red; N/A—not available.

In tests performed during one growing season on five GBV-1-infected grapevine accessions from a grapevine virus collection, the virus was not detected by real-time PCR in the shoots of two vines (PMC-022 and PMC-313) at the beginning of the growing season (April), and month later from the leaf petioles of grapevine accession PMC-022. During the other sampling periods, which included the period of dormancy, GBV-1 was successfully detected using either petioles (vegetation) or cortical scrapings (dormancy) from all five grapevine accessions, with the exception of accession PMC-313 which was not tested in March because of sample deterioration due to inadequate storage conditions (Figure 2).

### 3.3. Virus Screening in Collection Plantations and Commercial Vineyards

Out of 4302 vines tested, 576 (~13.4%) were positive for GBV-1. Of this, 41 (~0.9%) were originating from collection plantations, while 535 (~12.4%) positive vines originated from commercial vineyards. In the continental region, the virus was detected only in Zagreb, in two grapevine collections: in 10 out of 196 vines (~5.1%) in the grapevine virus collection and additional 15 out of 113 vines (~13.3%) originating from a national grapevine collection located at experimental station “Jazbina”. The presence of GBV-1 was not confirmed in the vines and rootstocks collection (91 vines), or in 441 tested vines from 16 different commercial vineyards in the continental region located in four counties, resulting in an overall infection rate in the continental region of ~1.9%.

**Figure 3:**
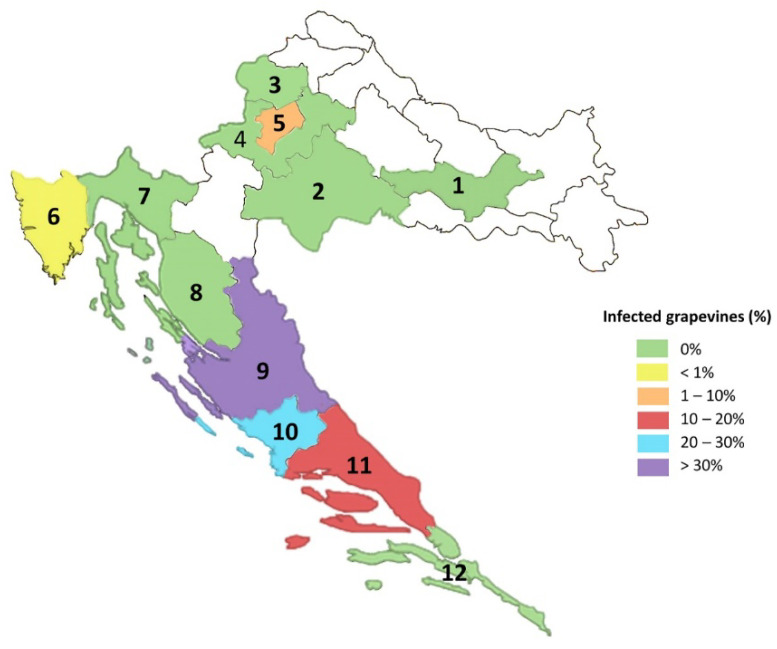
Distribution of GBV-1 throughout the Croatian counties by percentage of infection; 1—Požega-Slavonia; 2—Sisak-Moslavina; 3—Krapina-Zagorje, 4—Zagreb County; 5—city of Zagreb; 6—Istria; 7—Primorje-Gorski Kotar; 8—Lika-Senj; 9—Zadar; 10—Šibenik-Knin; 11—Split-Dalmatia; 12—Dubrovnik-Neretva. Other counties, shown in white, were not included in the survey.

In the coastal region, GBV-1 was detected in the collection plantation in Split in 16 out of 105 vines (~15.2%). In addition, 2878 samples collected from 72 different commercial vineyards/locations from seven counties were tested and 535 (~18.6%) originating from 30 locations (~41.7%) were positive for GBV-1. Looking at positions individually, the highest incidence of GBV-1 was found at Queen’s beach (Nin) and Ivan Dolac 1 (island of Hvar), where 48 out of 50 vines tested (96%) were positive. Most of the samples collected from commercial vineyards in the coastal region were from cv. Plavac mali—PMC (461), of which 142 (~30.8%) were infected. Considering only infections determined in the collection plantation and commercial vineyards, the overall GBV-1 infection rate determined in the coastal region was ~18.5%. Finally, out of 12 counties included in the survey, presence of GBV-1 was confirmed in five: city of Zagreb 25/878 (~2.8%); Istria 2/355 (~0.6%); Zadar 285/727 (~39%); Šibenik-Knin 11/40 (~27.5%), and Split-Dalmatia 253/1570 (16%) (Figure 3). A detailed overview of GBV-1 positive samples with corresponding locations is given in Table 2, while information on regions, locations, number of samples and cultivars included in survey can be found in the Supplementary Table S2.

### 3.4. Direct Sequencing and Phylogenetic Analysis

After conventional PCR, 50 newly discovered GBV-1 isolates were Sanger sequenced in both directions and submitted to GenBank under the accession numbers OM320482-OM320531. After the primer’s removal, sequences of 375 nts in length revealed 277 conserved, 98 variable, and 52 parsimony-informative sites, whereas amino acid sequences consisted of 124 amino acids with 111 conserved, 13 variable, and 4 parsimony-informative sites (Supplementary Table S3). Their nucleotide and amino acid identities ranged from 94.1 to 100% and from 92.8 to 100%, respectively.

**Figure 4:**
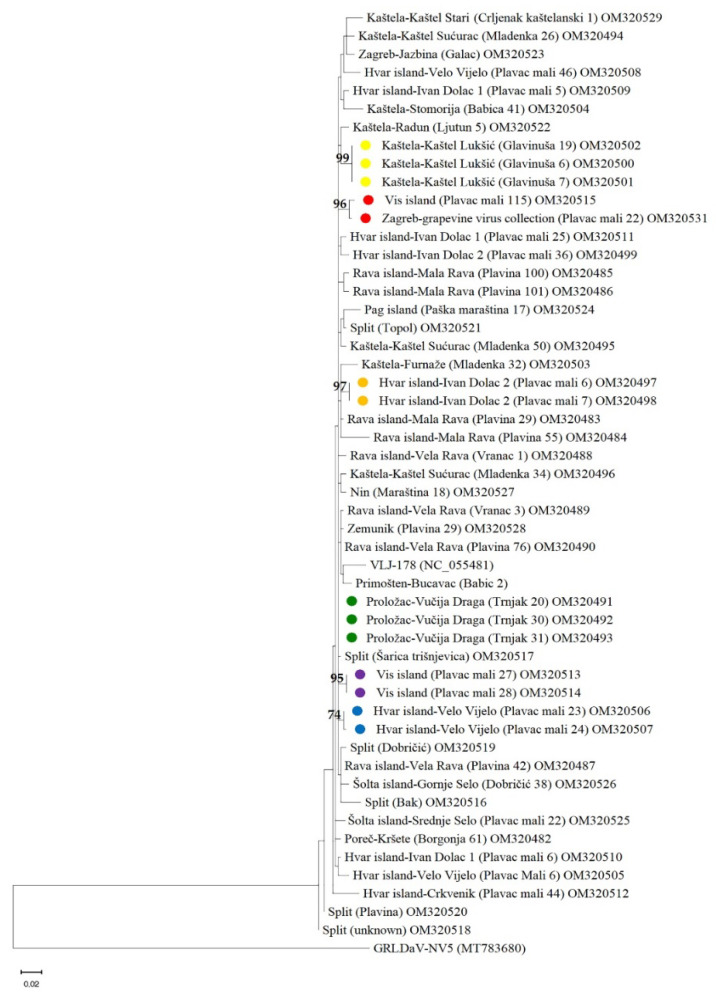
Maximum Likelihood (ML) tree showing phylogenetic relationships based on a 375 nts long sequences of the reverse transcriptase (RT) coding region of 50 newly discovered GBV-1 isolates, the reference isolate VLJ-178 and the grapevine Roditis leaf discoloration-associated virus (GRLDaV) isolate NV5 (MT783680) as a rooting outgroup. The tree was constructed using MEGA11 with the Tamura 3-parameter + gamma distribution (T3 + G) model of nucleotide substitution. Isolates were named according to the region, location of the vineyard, cultivar and exact vine/sample. Isolates marked with colored dots are collected from the same vineyard/collection and are represented on branches with a support greater than 50%.

Phylogenetic analyses performed using a ML tree revealed six cases of isolates clustering together, which originated from the same vineyards (Kaštela -Kaštel Lukšić, Visisland and Zagreb-grapevine virus collection, as the plant from Zagreb originated from the same vineyard on the island of Vis, Hvar island—Ivan Dolac 2, Proložac-Vučija Draga, Vis island, Hvar island—Velo Vijelo)). All other clusters formed with branch support below 50% (Figure 4). In other words, there was limited evidence of genetic separation among GBV-1 isolates in Croatia.

## 4. Discussion

So far, studies on grapevine viruses in Croatia have been carried out mainly on viruses whose detrimental effect on grapevine has been demonstrated and documented for a long time [26,33,34,35]. This study on GBV-1 was the first large-scale testing conducted on a recently discovered virus in Croatia with limited information concerning all other aspects except genome data and symptoms on limited number of vines with mixed virus infections.

In the validation and sensitivity comparisons of the PCR assays developed here (real-time and conventional) using two different DNA extraction methods (GES and DNeasy), real-time PCR was found to be 1000-fold more sensitive compared with conventional PCR for both isolation methods. Detection using column-based DNA extraction by Dneasy Plant Mini Kit was 100-fold more sensitive compared to detection using GES method. However, the concentration and purity of isolated DNA by GES was always satisfactory for real-time PCR, which was also confirmed by tests on five grapevine accessions in three replicates (Supplementary Figure S2D).

Virus detection during dormancy and the growing season showed that the beginning of the season (April, May) can lead to false-negative results, as noted for a subset of grapevine accessions. Similar results have been reported for grapevine leafroll-associated virus 3 in the USA and Canada, where virus concentration varied significantly with month of sampling and virus titer increased until June and decreased thereafter [36], or where the detection rate in May was also very low and virus titer increased until September [37]. In addition, it was not possible to detect viruses involved in grapevine leafroll disease by ELISA using leaves before inflorescences were fully developed [38]. Similarly, the study on GRBV dynamics showed a false-negative result when sampling was taken during the early growing season [39]. In contrast, studies on grapevine fanleaf virus (GFLV) and GPGV showed higher virus titers at the beginning of the growing season (May and June) compared to later sampling period [40,41,42]. All of this suggests that the ability of viruses to translocate to and remain in a particular tissue varies widely, so the ability to detect them varies greatly [41].

Screening of 4302 grapevine samples from different wine-growing regions confirmed a GBV-1 incidence of 13.4%, supporting the fact that the occurrence is not uncommon, but comparable to other viruses considered economically important and confirmed in Croatia, particularly GFLV [5,43,44]. The overall infection rate determined in the coastal region was much higher compared to the continental region, with an infection rate between 20 and 30% in Šibenik-Knin and over the 30% in Zadar County, while commercial vineyards in the continental region were free of GBV-1 (Figure 3). The significant difference between these two regions may be related, especially in case of some autochthonous cultivars, to the limited sources used for the production of planting material and the practice of on-site grafting, which is usually performed on the existing/old rootstocks. In fact, the presence of GBV-1 in the continental region was restricted only to vines from two collection plantations and, again, detected only in cultivars which origins are from the coastal wine-growing region. The prevalence of GBV-1 determined in this study is comparable to the frequency of another badnavirus infecting grapevine, GVCV, which was found in 8% of 1600 grapevines analyzed in the USA state of Missouri [45]. The largest number of samples from commercial vineyards was collected from cv. Plavac mali, the most important autochthonous, red-berried cultivar in Croatia, with determined infection rate of 30.8%. Moreover, the high infection rate of this cultivar with economically important viruses is already known [26,44], which is a major challenge in clonal selection. However, GBV-1 was not detected in autochthonous cvs. Škrlet, Belina starohrvatska, Pošip, Malvasia, Teran, Žlahtina, Kujundžuša, Vlaška and Malvasia dubrovačka, as well as in introduced cvs. Rhein Riesling, Müller-Thurgau, Pinot noir, Pinot gris, Gewürztraminer, Blauer Portugieser, Centennial seedless, Chardonnay, Merlot, Muscat and Cabernet Sauvignon, and Graševina as cultivar with uncertain origin.

High infection rates, especially in the location of Queen’s beach (96%) where half of the infected vines were from an old part of the vineyard and the other half was from newly planted vines, suggested the possibility of insect transmission. Literature indicates that some members of the genus *Badnavirus* are successfully transmitted by mealybugs [12], vectors that also successfully transmit several economically important grapevine viruses from the leafroll and rugose wood complexes [46,47].

The phylogenetic analysis, although conducted using relatively short portion of the conserved RT region, showed several interesting things. As shown in Figure 4, in six cases isolates from the same sites were grouped close to each other with more than 50% branches support, suggesting that they are genetically very similar or even identical and have a potentially recent common ancestor, or indicating the possibility of on-site insect vector-mediated transmission. In general, phylogenetic analyses confirmed limited evidence of genetic separation and spatial structuring, which, as noted above, could be the result of limited number of mother plants used for propagation, on-site grafting common in the coastal region, and movement of contaminated material across the country. This type of long-distance spread by vegetative propagation has been reported previously for other badnaviruses [14], but should also be investigated for GBV-1.

After the discovery of GBV-1 in grapevine samples from Croatia in 2018 [22], and the recent finding in 2022 [26], Croatia remains, to our knowledge, the only country where the virus has been confirmed, and apart from GBV-1 genomic data, nothing else has been reported. Nevertheless, this study is evidence of the wide distribution of GBV-1 in the Croatian coastal wine-growing region. This could be important since several species of badnaviruses are known to cause economically important losses in their tropical hosts, which can be as high as 90% (i.e., banana streak virus in bananas, citrus yellow mosaic badnavirus in citruses and cacao swollen shoot virus in cocoa) [13]. Economic damage caused by badnaviruses affects not only tropical crops but also grapevines, as some vineyards in the USA have been uprooted due to GVCV infections [14,15]. In addition, GRLDaV, the other badnavirus affecting grapevine, has been placed on the EPPO alert list as potentially dangerous, although very littledata isavailable on its biology, epidemiology, distribution, and impact on grape production [25]. Our further studies will focus on a better understanding of the ecology, epidemiology, and cytopathology of GBV-1 and its impact on grapevine performance.

## 5. Conclusions

This study contributed to the development of robust and reliable PCR-based detection methods (real-time and conventional) for GBV-1, which are useful tools for grapevine virus diagnostics, epidemiological studies and certification schemes. Through large-scale testing we have confirmed the frequent occurrence of GBV-1, especially in the Croatian coastal wine-growing region. Phylogenetic analyses clustered some isolates collected at the same site together, suggesting recent common ancestry and possible local spread, but at the country level the virus is spread by contaminated planting material.

## Figures and Tables

**Table 1:** Primers and probes used for grapevine badnavirus 1 (GBV-1) detection in real-time and conventional PCR assays.

Target	Primer	Orientation	Assay/Target Gene	Primer/Probe Sequence (5′–3′)	Product Size (bp)
GBV-1	GBV-1-F	Forward primer	Real-time PCR/reverse transcriptase (RT)	GGYAAGGAAAGAATGGTCTTCA	181
	GBV-1-R	Reverse primer		TCCATTCTATAGAATCTGGGTGCAT	
	GBV-1-P	TaqMan probe		AAGATCAATATAGCCTTCCTGGA	
	GBV-1-F	Forward primer	Conventional PCR/reverse transcriptase (RT)	GGYAAGGAAAGAATGGTCTTCA	419
	GBV-1-R_con	Reverse primer		TTTGTTGGGCTCARGACAAGCC	

**Table 2:** Number of GBV-1-positive grapevines identified by real-time PCR. For each positive location details concerning the location and type of vineyards (commercial or collection) are given. Only locations where presence of GBV-1 was confirmed are shown.

Wine-Growing Region	County	Region	Location	Positive/Total Number of Analysed Samples (%)
**Continental**	city of Zagreb	Zagreb	Grapevine virus collection	10/196 (5.1%)
			Jazbina (National collection 2)	15/113 (13.3%)
**Coastal**	Istria	Poreč	Kršete	2/105 (1.9%)
	Zadar	Rava island	Mala Rava	66/194 (34%)
			Vela Rava	85/110 (77.3%)
		Pag island	Pag 2	10/30 (33.3%)
			Pag 3	32/58 (55.2%)
			Pag 4	15/30 (50%)
		Nin	Queen’s beach	48/50 (96%)
		Zemunik	Zemunik Donji	29/50 (58%)
	Šibenik-Knin	Primošten	Vezac	4/10 (40%)
			Bucavac	7/30 (23.3%)
	Split-Dalmatia	Kaštela	Kaštel Sućurac	6/50 (12%)
			Radun	8/54 (14.8%)
			Kaštel Lukšić	22/50 (44%)
			Furnaže	9/50 (18%)
			Stomorija	2/50 (4%)
			Kaštel novi	7/70 (10%)
		Split	Collection plantation	16/105 (15.2%)
		Proložac	Vučija Draga	5/135 (3.7%)
		Hvar isand	Velo vijelo	12/50 (24%)
			Ivan Dolac 1	48/50 (96%)
			Ivan Dolac 2	34/50 (68%)
			Crkvenik	24/50 (48%)
		Vis island	Petričevo	32/231 (13.8%)
		Šolta island	Srednje selo	19/130 (14.6%)
			Gornje selo	9/51 (17.6%)

## Data Availability

All sequencing data of Croatian GBV-1 isolates obtained in this research were included in the manuscript and/or submitted to GenBank database. Accession numbers: OM320482-OM320531.

## Supplementary Materials

The following are available online at www.mdpi.com/article/10.3390/plants11162135/s1, Figure S1: Croatian counties included in survey. Figure S2: Sensitivity comparison between real-time PCR and conventional PCR assays for the GBV-1 detection taken on grapevine accession PMC-313 using three replicates of 10-fold dilution series and DNA isolated using GES. Table S1: Sequences of four newly discovered GBV-1 isolates determined by HTS and used, together with reference isolate NC_055481, for development of primers and probes for real-time and conventional PCR. Table S2: Number of collected and GBV-1-positive vines included in the study of GBV-1 distribution by counties, regions and vineyard locations in continental and coastal part of Croatia. Table S3: Multiple sequence alignment of the partial reverse transcriptase (RT) genes of 375 nts (124 aa) long sequences from 50 newly detected GBV-1 isolates and reference isolate VLJ-178.
