# Supplementary material for: Grapevine Badnavirus 1: Detection, Genetic Diversity, and Distribution in Croatia
Source: Plants (Basel). 2022 Aug 16;11(16):2135. doi: 10.3390/plants11162135 (PMC9416389; doi:10.3390/plants11162135)
Supplement: Supplementary file 1 [file plants-11-02135-s001.zip › Supplementary Figure S1.pdf]

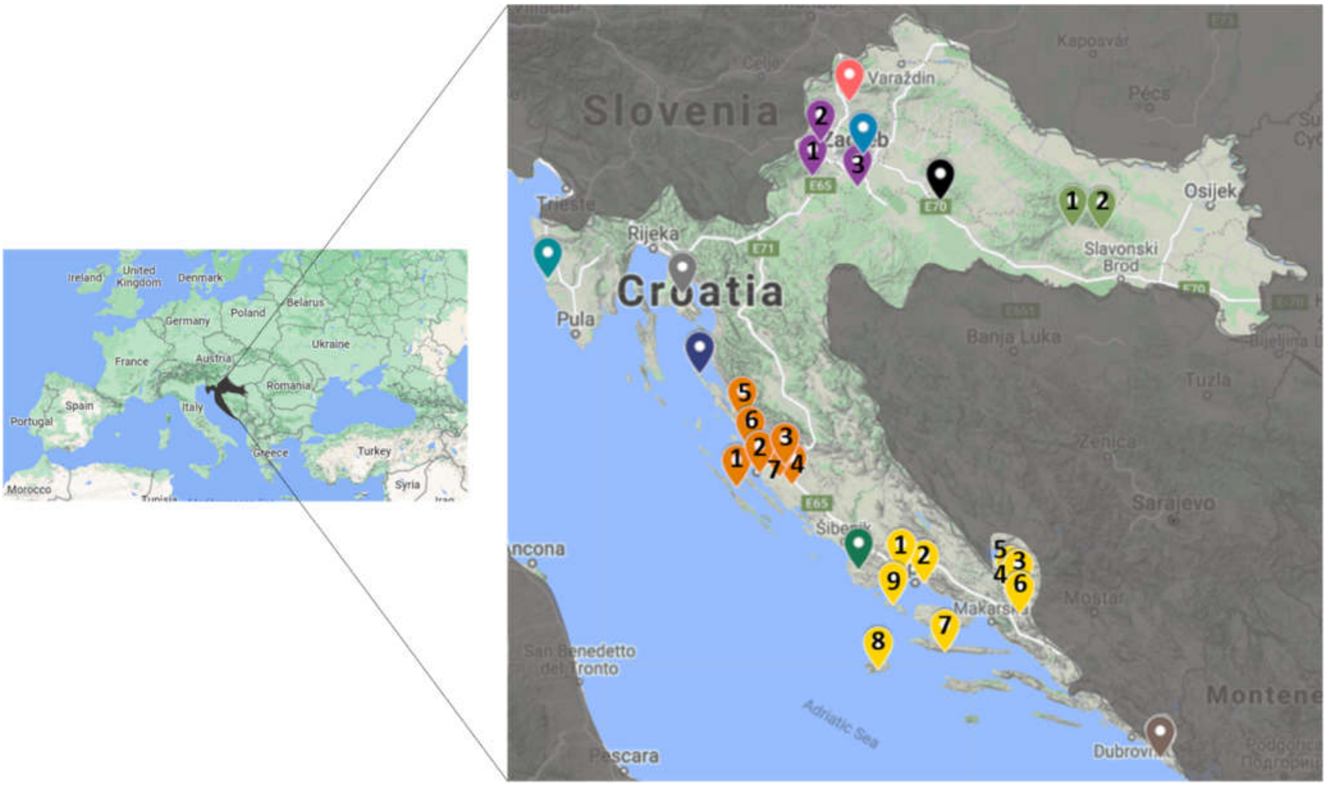

Supplementary Figure S1. Croatian Counties included in survey: **green marks** – Požega-Slavonia: 1-Velika, 2-Kutjevo; **black mark** - Sisak-Moslavina: Popovača; **red mark** - Krapina-Zagorje: Sveti Križ Začretje; **purple marks** – Zagreb county: 1-Plešivica, 2-Marija Gorica, 3-Velika Gorica; **blue mark** – Zagreb; **turquoise mark** – Istria: Poreč; **grey mark** – Primorje-Gorski Kotar: island of Krk ; **dark blue mark** – Lika-Senj: island of Pag; **orange mark** – Zadar: 1-island of Rava, 2-Zadar, 3-Poličnik, 4-Benkovac, 5-island of Pag , 6-Nin, 7-Zemunik; **dark green mark** – Šibenik-Knin: Primošten; **yellow marks** – Split-Dalmatian: 1-Kaštela, 2-Split, 3-Imotski, 4-Podbablje, 5-Proložac, 6-Runovići, 7-island of Hvar , 8-island of Vis, 9-island of Šolta; **brown mark** - Dubrovnik-Neretva: Konavle.
