# Supplementary material for: Grapevine Badnavirus 1: Detection, Genetic Diversity, and Distribution in Croatia
Source: Plants (Basel). 2022 Aug 16;11(16):2135. doi: 10.3390/plants11162135 (PMC9416389; doi:10.3390/plants11162135)
Supplement: Supplementary file 1 [file plants-11-02135-s001.zip › Supplementary Figure S2.pdf]

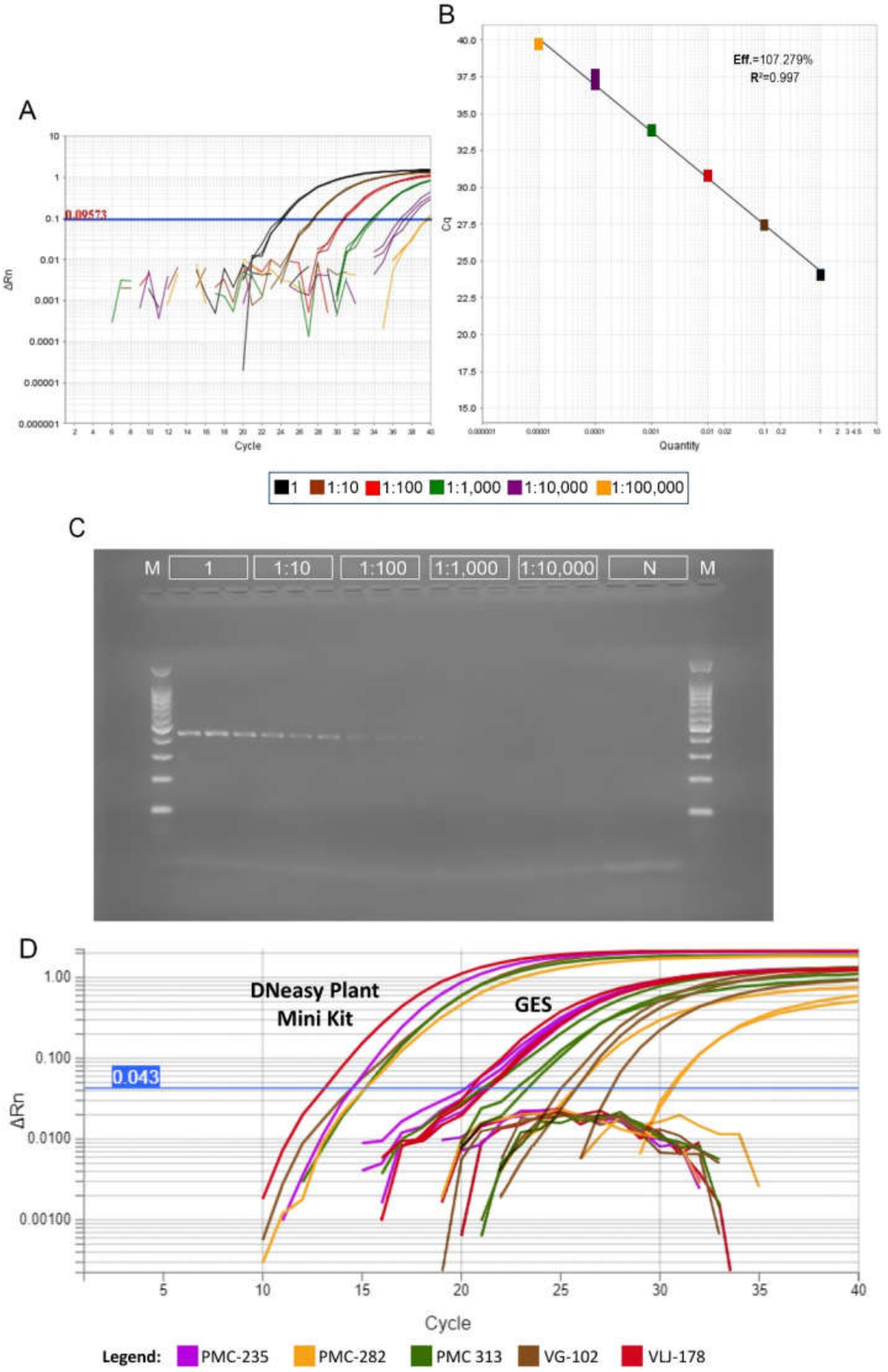

Supplementary Figure S2. Sensitivity comparison between real-time PCR and conventional PCR assays for the GBV-1 detection done on grapevine accession PMC-313 using three replicates of 10-fold dilution series. DNA was isolated using the GES extraction method [29]. 1 - undiluted extract; 1:10 - 1:100,000 - serial 10-fold dilutions. **(A)** Plots of DNA dilution series against threshold cycles values showing the dynamic range of the real-time PCR assay detection. The lines below threshold represents negative controls for each dilution. **(B)** Standard curve analysis of the real-time PCR sensitivity: x-axis - DNA dilution; y-axis - measured quantification cycle (Cq) value; R2 - determination coefficient; Eff.- real-time PCR efficiency. **(C)** PCR products obtained by conventional PCR on a 1.5% TBE agarose gel; M-marker (GelPilot 100 bp Plus Ladder, Qiagen, Hilden, Germany), N – negative controls for undiluted extract. **(D)** Comparison of amplification curves obtained by real-time PCR between five grapevine accessions (PMC-235, PMC-282, PMC-313, VG -102, VLJ-178), whose DNA was extracted with the DNeasy Plant Mini Kit and with the GES method. The broken lines below the threshold represents negative controls for each grapevine accession.
